# Determining a low disease activity threshold for decision to maintain disease-modifying antirheumatic drug treatment unchanged in rheumatoid arthritis patients

**DOI:** 10.1186/ar2836

**Published:** 2009-10-23

**Authors:** Michel de Bandt, Bruno Fautrel, Jean Francis Maillefert, Jean Marie Berthelot, Bernard Combe, René-Marc Flipo, Frédéric Lioté, Olivier Meyer, Alain Saraux, Daniel Wendling, Xavier Le Loët, Francis Guillemin

**Affiliations:** 1 Centre hospitalier d'Aulnay sous Bois, Service de Rhumatologie, Boulevard Ballanger, Aulnay sous Bois F-93600, France; 2 APHP-GH Pitié Salpêtrière, Service de Rhumatologie, UFR de Médecine, Université Paris VI - Pierre et Marie Curie, 83 boulevard de l'Hôpital, 75651 Paris cedex 13, France; 3 Centre Hospitalo-Universitaire du Dijon, Hôpital du Bocage, Service de Rhumatologie, 3 rue du faubourg Raynes, Dijon F-21000, France; 4 INSERM ERI 7 (EA 3822), Centre Hospitalo-Universitaire de Nantes, Hotel-Dieu, Service de Rhumatologie, 1 Place Alexis Ricordeau, Nantes F-44000, France; 5 Centre Hospitalo-Universitaire du Montpellier, Hôpital Lapeyronie, Service de Rhumatologie, 371 avenue du Doyen Gaston Giraud, Montpellier F-34000, France; 6 Centre Régional Hospitalo-Universitaire de Lille, Service de Rhumatologie, Rue du Pr E Laine, Lille F-59000, France; 7 Hôpital Lariboisière, Centre Viggo-Petersen, Service de Rhumatologie, 2 rue A Paré, Paris F-75010, France; 8 UFR de Médecine - Bichat Lariboisière, Université Paris 7, APHP, Groupe hospitalier Bichat - Claude Bernard, Service de Rhumatologie, 46 rue H Huchard, Paris F-75018, France; 9 Centre Hospitalo-Universitaire de Brest, Hôpital de la Cavale Blanche, Service de Rhumatologie, rue T Prigent, Brest F-29000, France; 10 EA3186 - Agents pathogènes et Inflammation, Université de Franche-Comté, Centre Hospitalo - Universitaire de Besançon, Hôpital Jean Minjoz, Service de Rhumatologie, 1 Bd Fleming, Besançon F-25000, France; 11 Department of Rheumatology, Rouen University Hospital & Inserm U905 (IFRMP 23), University of Rouen, 1 rue de Germont, Rouen F-76230, France; 12 INSERM CIC-EC, CHU de Nancy - Hôpital Marin, 92 av Mal de Lattre de Tassigny, 54035 Nancy cedex, France; 13 Université Henri Poincaré Nancy I, EA4003, Ecole de Santé Publique, Faculté de Médecine de Nancy, Nancy F-54000, France

## Abstract

**Introduction:**

The aim of this study was to determine a low disease activity threshold - a 28-joint disease activity score (DAS28) value - for the decision to maintain unchanged disease-modifying antirheumatic drug (DMARD) treatment in rheumatoid arthritis patients, based on expert opinion.

**Methods:**

Nine hundred and sixty-seven case scenarios with various levels for each component of the DAS28 (resulting in a disease activity score between 2 and 3.2) were presented to 44 panelists. For each scenario, panelists had to decide whether or not DMARD treatment (excluding steroids) could be maintained unchanged. In each scenario, for decision, the participants were given the DAS28 parameters, without knowledge of the resultant DAS28. The relationship between panelists' decision, DAS28 value, and components of the score were analysed by multiple logistic regression analysis. Each panelist analysed 160 randomised scenarios. Intra-rater and inter-rater reproducibility were assessed.

**Results:**

Forty-four panelists participated in the study. Inter-panelist agreement was good (κ = 0.63; 95% confidence interval = 0.61 to 0.65). Intra-panelist agreement was excellent (κ = 0.87; 95% confidence interval = 0.82 to 0.92). Quasi-perfect agreement was observed for DAS28 ≤ 2.4, less pronounced between 2.5 and 2.9, and almost no agreement for DAS28 > 3.0. For values below 2.5, panelists agreed to maintain unchanged DMARDs; for values above 2.5, discrepancies occurred more frequently as the DAS28 value increased. Multivariate analysis confirmed the relationship between panelist's decision, DAS28 value and components of the DAS28. Between DAS28 of 2.4 and 3.2, a major determinant for panelists' decision was swollen joint count. Female and public practice physicians decided more often to maintain treatment unchanged.

**Conclusions:**

As a conclusion, panelists suggested that in clinical practice there is no need to change DMARD treatment in rheumatoid arthritis patients with DAS28 ≤ 2.4.

## Introduction

The aim of rheumatoid arthritis (RA) treatment is remission. The combination of new potent treatments, early intervention, and a treatment strategy of tight control makes remission a daily possibility [1-3]. In clinical practice, active disease means that clinicians will increase the treatment to obtain remission; but that also means that clinicians can stop, add to or increase treatments once the goal is achieved. What is the level of activity to amend treatment?

Even with a combination of multiple therapeutic agents, drug-induced remission is difficult to reach as Pinals criteria [4] are difficult to achieve in clinical trials, even when using the most potent new drugs. A better alternative might therefore be to change the remission criteria to other criteria.

For this purpose a new therapeutic goal has been proposed. A state of near remission or partial remission, or of minimal disease activity or low disease activity, has been proposed in recent years, to be used in clinical trials or guidelines with new disease-modifying antirheumatic drug (DMARD) treatments arriving on the market in the 2000s [3,5-11].

This area of low disease activity might be limited by two thresholds, an upper threshold and a lower threshold, which are set to help the treatment decision. The upper threshold of disease activity is the level above which treatment prescription in naïve patients or a switch/intensification in previously treated patients is needed and strongly recommended, and the lower threshold is the level below which treatment maintenance could be recommended; some uncertainties remain for practical issues between these two thresholds.

Persistence of moderate to high disease activity (whatever the measurement criteria we use) is the usual standard to decide whether treatment should be considered ineffective and patients should be switched to another treatment. But on the contrary, when treatment is effective, even with persistence of some degree of low disease activity, the question remains how to decide whether it is worth not changing and better maintaining current treatment, to spare further resources.

Several disease activity measures are currently available, and indices such as the 28-joint disease activity score (DAS28) or derivates are currently used for such decisions at the upper bound. Usually DAS28 > 3.2 is accepted to consider the disease active enough and to reinforce the treatment dosage or to switch the DMARD [5-12]. On the contrary, DAS28 < 2.4 defines remission, but there is no recommendation telling the clinician what to do once this level is reached.

Some uncertainties still remain in defining the lower threshold below which treatment maintenance could be recommended. The first uncertainty is that there is no agreement on the best tool to be used to define this state of near remission. Practically, the DAS28 remains the most used in clinical practice and has been validated is trials - with some areas of uncertainty below the threshold of indication for changing therapy (3.2) and around the level of remission (2.6) as proposed by the DAS28 designers. The second issue is that the definition of such low disease activity implies the recognition of a threshold value of activity under which the disease could be considered low enough to keep treatment unchanged (except steroids), and therefore serves as a basis for clinicians to maintain unchanged the treatment.

As everyday practice is different from clinical trials, we aimed to determine such a threshold based on the expert physicians' decision method and to develop recommendations for clinicians to use the DAS28 in clinical practice in the area of uncertainty (DAS28 of 2 to 3.2), in order to guide the decision to maintain unchanged or to not maintain a DMARD treatment; that is, to avoid changing because of sufficient/acceptable effectiveness.

The present study was designed to determine a low disease activity threshold - that is, a DAS28 value - below which DMARD treatment should not be changed in RA patients.

## Materials and methods

### Design

The present survey was conducted using paper case scenarios of patients with various levels of disease activity presented to rheumatologists in public and private practice.

### Sample

The stratégie thérapeutique de la polyarthrite (STPR) initiative is a group of rheumatology experts from French university hospitals (public) working on implementation of practical guidelines for therapeutic strategy in RA [13-15]. Each STPR expert recruited rheumatologist physicians with exclusive private or public practice or with combined practice among rheumatologists trained by the STPR group in each region of France to understand and implement current recommendations of treatment. A group of 44 panelists was constituted for this work.

### Case scenarios

Virtual scenarios were constructed on a distribution of DAS28 values (four variables) in steps of 0.1 from 2.0 to 3.2 - chosen to be below the proposed threshold of indication for changing therapy (3.2) and around the level of remission (2.6) [1-3] - among all possible combinations of DAS28 parameters: erythrocyte sedimentation rate (ESR), patient global activity, number of swollen joints, and number of tender joints.

To limit the number of possible scenarios with all possible combinations resulting in DAS28 values within the range of 2.0 to 3.2, it was decided to increment the DAS28 by 0.1 points for assessment of disease activity, by 5 points from 10 to 50 for the ESR (mm/hour), and by 1 point from 0 to 28 for the number of tender joints and for the number of swollen joints, thus constituting 967 case scenarios.

All case scenarios were presented considering that RA activity had been stable for the past 3 months. The participants were given the DAS28 parameters, without knowledge of the resultant DAS28 value. For example, a scenario corresponding to a DAS28 of '*s*' (not provided to the respondent) was 'In this patient with clinical status stable since 3 months with ESR = *x*, number of tender joint = *y*, number of swollen joint = *z *and global assessment of disease activity = *t*, would the level of disease activity be considered low enough for maintaining the current DMARD therapy (except steroids that were not considered as DMARDS in this work) without changing neither molecule nor dose regimen? Answer: Yes or No.'

Interviewees were instructed to consider this clinical status, without any specific joint involvement and whatever the current therapy, including corticosteroid or not, and were not asked to calculate the disease activity score.

### Reproducibility design

Intra-panelist reproducibility and inter-panelist reproducibility were assessed and the determinant of decision was analysed.

Inter-rater reproducibility was evaluated. A first set of 60 scenarios in the range 2.0 to 3.2 (five scenarios in each of the 12 steps of 0.1) was obtained randomly and submitted to the 13 STPR experts. The total 60 scenarios were presented in random order, and were answered by self-administration.

Intra-rater reliability was evaluated during the next step (survey, see below). A set of 12 scenarios was randomly selected among the 967 cases. The scenarios were duplicated and these 24 scenarios were mixed randomly within all the scenarios submitted to the panelists.

### Survey

All panelists were asked to rate the 967 case scenarios. In order to increase the feasibility, it was decided that participants would not have to rate all scenarios. Therefore 184 scenarios were given to every participant; that is, 160 out of 955 (967 - 12) scenarios were randomly attributed, which differed from one rheumatologist to another (each random choice conducted using the proc plan procedure in SAS software; (SAS Software, Cary NC, USA), and were mixed with the 24 (12 × 2) scenarios described above for evaluation of intra-rater reliability.

A research nurse conducted the rating interview with the panelist on the telephone as a support. Panelists and nurses had the same material of case scenarios available. The survey was completed within a 4-week period from May to June 2007.

### Statistical analysis

#### Reproducibility analysis

Inter-panelist reliability was assessed by the multirater κ coefficient [16] and its 95% confidence interval (CI), which ranges from 0 to 1 and rates the agreement between raters as low (< 0.2), fair (0.2 to 0.4), moderate (0.4 to 0.6), good (0.6 to 0.8) or excellent (> 0.8).

Results are also presented considering perfect agreement (restricted to all experts making the same decision) and considering quasi-perfect agreement (accepting 80% of experts making the same decision). The determinants of agreement between panelists among the components of the DAS28 were sought by logistic regression analysis of perfect agreement and of quasi-perfect agreement. Odds ratios were considered significant at α = 0.05.

Intra-panelist reliability was assessed by Cohen's κ coefficient for agreement. The probability (odds ratio) of determinants of agreement within panelists was assessed using logistic regression.

#### Determining threshold for treatment maintenance

The proportion of panelists' opinions to maintain or change treatment regimen was expressed as a mean percentage of answers to scenarios at each step of the DAS28 value.

The determinants of the decision to change the treatment regimen were further analysed in a logistic regression model where odds ratios were considered significant at α = 0.05. This analysis was conducted over all answers to scenarios and was replicated in each group of scenario by steps of 0.1 points of DAS28 values. Because the number of case scenarios increased by category, a subset of 310 answers to scenarios was randomly selected in each step category to allow similar comparison of model results and interpretation in terms of power and significance of the conclusion reached.

All analyses were conducted under SAS software version 9.1.

The results of the whole analysis were then examined by the STPR group in order to edit recommendations for the decision to maintain DMARD treatment unchanged in RA patients.

## Results

### Experts

A total of 44 panelists participated in the study, including 13 experts or members from the STPR group. They were on average 43 ± 6.6 years old, 38% were female, and they obtained their certification as rheumatologists on average 17 ± 6.3 years ago. The panelists' clinical activity was university public hospital (65%) or private practice (35%).

### Inter-panelist reliability and intra-panelist reliability

Among the 60 scenarios presented to panelists, 59 were filled in by the 13 experts of the STPR group and the agreement reached a κ value of 0.63 (0.61 to 0.65). The κ value increased to 0.69 (0.65 to 0.73) and 0.76 (0.72 to 0.80) when restricting the analysis to the 12 most concordant and the 11 most concordant panelists, respectively.

**Figure 1:**
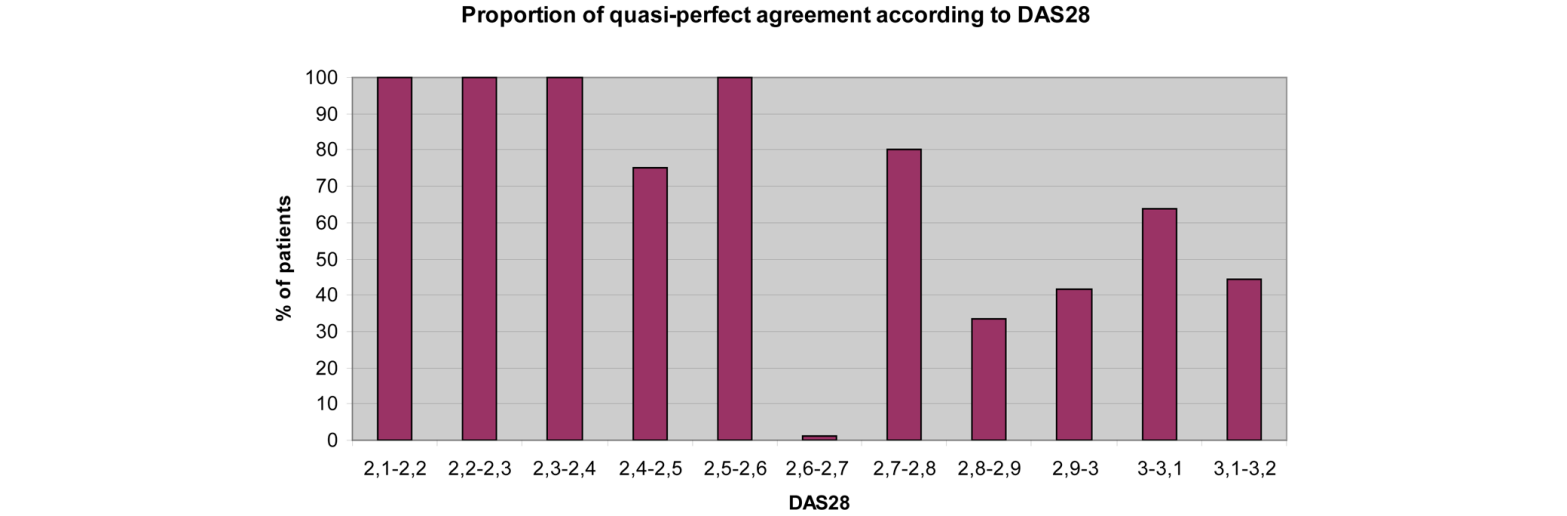
Proportion of quasi-perfect agreement according to 28-joint disease activity score. Distribution of cases by 28-joint disease activity score (DAS28) value (incremented in steps of 0.1 from 2.0 to 3.2) and the percentage of quasi-perfect agreement decreasing over a DAS28 value of 2.6.

Further reduction did not modify κ values meaningfully. The inter-rater agreement was therefore good. Figure 1 shows the distribution of cases by DAS28 value with quasi-perfect agreement over a value of 2.6.

The probability of inter-panelist quasi-perfect agreement was significantly higher when the patient global evaluation of activity, the ESR and the number of swollen joints were higher (Table 1).

**Table 1:** Determinants of inter-panelist perfect and quasi-perfect agreement

	Perfect agreement		Quasi-perfect agreement	
	
	Odds ratio	95% confidence interval	Odds ratio	95% confidence interval
Patient global assessment of disease activity	1.01	0.97 to 1.05	1.06	1.01 to 1.12
Erythrocyte sedimentation rate	1.05	0.99 to 1.12	1.15	1.04 to 1.27
Number of tender joints	0.88	0.41 to 1.88	3.00	1.23 to 7.31
Number of swollen joints	0.99	0.88 to 1.11	-^a^	

The probability of inter-panelist perfect agreement was significantly higher when the patient global evaluation of activity, erythrocyte sedimentation rate and number of swollen joints were higher. ^a^Not included in the model because of quasi-complete separation of the data point.

Intra-rater reliability was excellent, with a κ value of 0.83 (95% CI = 0.78 to 0.88).

### Threshold for treatment maintenance

**Figure 2:**
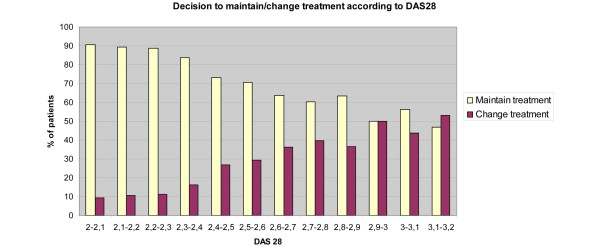
Decision to maintain/change treatment according to 28-joint disease activity score. Proportion of cases by category of 28-joint disease activity score (DAS28) (range 2.0 to 3.2) analysed with a decision to maintain the treatment from 7,224 answers. Panelists were in quasi-agreement to make a decision to maintain treatment in more than 80% of cases for DAS28 < 2.4, decreasing to 70% until a DAS28 of 2.6, and around 60% for DAS28 > 2.9.

The decision to maintain treatment was analysed from 7,224 answers. Figure 2 shows the proportion of cases by category of DAS28 value with the decision to maintain treatment. Panelists were in quasi-agreement to make a decision to maintain treatment in more than 80% of cases for DAS28 < 2.4, decreasing to 70% until a DAS28 value of 2.6, and then around 60% from DAS28 > 2.9.

Overall, all DAS28 components were significant determinants of the decision to maintain treatment unchanged (Table 2). Higher patient global assessment of disease activity, ESR, number of tender joints and number of swollen joints reduced significantly the likelihood of the decision to maintain treatment unchanged. For instance, a patient with global activity of 15, ESR of 10, no tender joint and 22 swollen joints would have a probability of treatment being unchanged of 2.54%. If one of each parameter increased by one unit, this would result in a lower probability of 2.48% with global activity of 16, a probability of 2.43% with ESR of 11, and a probability of 2.04% with 23 swollen joints or one tender joint.

**Table 2:** Determinants of maintaining treatment unchanged

	Odds ratio	95% confidence interval	*P *value
Patient's global assessment of disease activity	0.15	0.11 to 0.21	< 0.0001
Erythrocyte sedimentation rate	0.49	0.37 to 0.65	< 0.0001
Number of tender joints	0.34	0.23 to 0.49	< 0.0001
Number of swollen joints	0.004	0.003 to 0.005	< 0.0001
Interaction (number of tender joints × number of swollen joints)	2.06	1.51 to 2.82	< 0.0001

Determined using multivariate logistic regression, n = 7,224. Overall, all 28-joint disease activity score (DAS28) components are significant determinants of the decision to maintain treatment unchanged. All variables are transformed according to the DAS28 formula.

Between DAS28 values of 2.4 and 3.2, each category increasing by 0.1 (310 randomly selected answers), the various regression analyses of the decision to change treatment showed that the major determinant for the panelists' decision to maintain treatment unchanged was the lower number of swollen joints (odds ratio from 0.4 (DAS28 between 2.4 and 2.5) to 0.7 (DAS28 between 3.0 and 3.1)).

Female (64% vs. 56%, *P *= 0.0002) and public practice physicians (61% vs. 50%, *P *< 0.0001) decided more often to maintain treatment unchanged. Decisions were not significantly related to panelists' characteristics of age (*P *= 0.6825) and year of diploma (*P *= 0.1124).

## Discussion

The present study has evidenced some major findings. The discordance in the decision to maintain or change treatment lies within the range of 2.5 to 3.2 for the DAS28 value, suggesting setting a consensual threshold of 2.4 to define maintenance of treatment. Second, panelists make the decision to maintain DMARD treatment (excluding steroids) with excellent intra-rater reliability, but only moderate to good inter-rater reliability. Also, determinants of the decision have been evidenced with remarkable persistence whatever the level of disease activity in this range; namely, the ESR level, the patient's global activity, the number of tender joints and the number of swollen joints are taken into consideration by experts to make their decision. Fourth, these results are independent of experts' characteristics of age, sex, and year of diploma, but not of type of practice. Finally, according to these results, panelists concluded that in clinical practice there is no need to change DMARD treatment (neither molecule nor dose regimen) when a patient with stable clinical status over the past 3 months has DAS28 ≤ 2.4.

Targeting remission is recommended given recent changes and innovations in therapy as well as the evolution of therapeutic strategies. There is no general consensus, however, regarding definition of remission. Achieving Pinals criteria (five out of six criteria: morning stiffness absent or not exceeding 15 minutes, no fatigue, no joint pain by history, no joint tenderness, no joint or tendon sheath swelling, and no elevation of the ESR) was estimated to be rare in the 1990s [4,10,11]. Moreover, Pinals criteria are difficult to apply in clinical trials.

The use of different definitions of RA remission leads to different results with regard to remission rates. Analysis of a database of more than 5,800 RA patients showed that the overall remission rate was lowest using the American College of Rheumatology definition of remission (8.6%), followed by the clinical disease activity index (13.8%) and routine assessment of patient index data (RAPID) 3 (14.3%) definitions; the rate was highest when remission was defined using the DAS28 (19.6%) [17].

Using the disease activity score, a state of near remission has been proposed recently [3,5,10,11] with two thresholds to help the treatment decision: an upper threshold of disease activity, above which intensification is recommended; and a lower threshold, below which treatment maintenance can be recommended. Usually, DAS28 > 3.2 is accepted to consider the disease active enough and to reinforce the treatment dosage or to switch DMARD [8]. The lower bound is less obvious and has been investigated using several methods [5-10]: the DAS28 value, the OMERACT definition, the patient acceptable symptom state (PASS) or the RAPID score (patient index data scores)

The value of 2.6 has been proposed by developers of the DAS28 [12]. After calculating the disease activity of patients included in a cohort as well as a modification of the American Rheumatology Association definition for clinical remission, receiver operating characteristic analysis was used to determine the cut-off point with maximum sensitivity and specificity in the DAS28 corresponding with fulfillment of the modified American College of Rheumatology criteria. The optimal cut-off value corresponding to American College of Rheumatology criteria for remission was 2.66 [7].

The disease activity score has been criticised for its performances in the spectrum of a low level of disease activity [11,18,19]. We found that panelists were in good agreement below a score of 2.5. Over this threshold, other data may be requested and may actually be fully incorporated in clinical practice judgment. Other patient-reported outcomes, in addition to the global patient assessment component, are also important to consider in weighing up the consequences of activity.

One limitation of the disease activity score remains that it does not specify whether swollen joints are ankles or metatarsophalangeal joints, possibly resulting in some unbalanced estimate of activity.

The OMERACT defined minimal disease activity as patients with no tender or swollen joints and ESR < 10 [20]. If this definition is not reached, patients must have either a DAS28 value ≤ 2.85 or meet five among seven criteria (pain ≤ 2, health assessment questionnaire score ≤ 0.3, tender joint count ≤ 1, swollen joint count ≤ 1, patient global activity ≤ 2, physician global activity ≤ 1.5, ESR ≤ 20).

As low disease activity is closely related to treatment decision-making, others have proposed to define a minimal clinically important improvement and a PASS. The concept of PASS translates the response at the group level (change in mean scores) into clinically meaningful information by addressing the patient level as therapeutic success (yes/no) in order to help the decision. The concept is not fully formulated at the present time [21,22].

Pincus and colleagues proposed a continuous quality improvement approach to assess and manage RA patients without formal joint counts, based on quantitative RAPID scores on a multidimensional health assessment questionnaire [23].

Furst and colleagues, using expert opinion and the Research and Development/University of California in Los Angeles (RAND/UCLA) Appropriateness Method, have proposed recently to consider that only the clinically active joint count should be considered the most important decision factor among a literature review of various outcome measures [24]. Among 108 scenarios developed to simulate various clinical situations, the panelists recommended that the clinically active joint count should be the most important decision factor, and in patients with no active joints they recommended that, regardless of other factors, treatment should not be changed. Patients with five or more active joints were considered inadequately treated, patients with no active joints had no need to change therapy, and in patients with one to four active joints other variables must be considered.

As some uncertainty still remains, we aimed to determine such a threshold based on the experts' decision method and to develop recommendations for clinicians in order to guide the decision to maintain unchanged or not a DMARD treatment; that is, to avoid changing because of sufficient/acceptable effectiveness. In this particular area below the threshold of indication for changing therapy (3.2) and at about the level of remission (2.6), we have shown that determinants of the decision have been evidenced with remarkable persistence whatever the level of disease activity in this range - namely, the ESR level, patient's global assessment of disease activity, number of tender joints and number of swollen joints are taken into consideration by experts to make their decision. The importance of patient-reported outcomes is now clearly acknowledged, and our results value both patients' and physicians' criteria.

Development of recommendations for clinical practice may use various methodologies and be data driven or expert driven. Elicitation of expert opinion uses the Delphi method, focus groups, consensus, and so forth. The data-driven methodology (DAS28) is based on the data observed and retrospectively analysed. The data are inherently subjected to indication bias (that is, results are dependent on the initial decision of treatment), which is driven by initial judgment of patient status - while expert opinions are dependent on the experts' choice (that is, representativity of experts is sometimes questionable). This latter observation may have some advantage in term of ease to collect data. Expert opinion is now largely developed and accepted as an alternative to produce consensus and informed decision.

One strength of the present work is that the judgments made were unbiased. The OMERACT method is largely used for reaching consensus, with its validated threshold proposed in the literature [21]. Our approach, however, asked experts to make their judgment without knowing the DAS28 value of scenarios - therefore, independently.

One important limitation of the present work is that it does not consider the long-term effect of maintaining low disease activity. Recent works have shown that even if patients are maintained under highly effective treatment, with low disease activity, the long-term deterioration of the joint continues to progress, tempering the enthusiasm of the new class of biologic agents. But in the context of spare resources, the aim is still to preserve the future possibility of action.

Another possible limitation is that we did not consider steroids as DMARDs, even if in light of recent data this opinion should be modified in the near future. We consider steroids useful at the very beginning of the disease, awaiting effectiveness of DMARDs or of local injection; but this is not the method utlilised by numerous colleagues. The last point (also shared with the disease activity score) is that the locations of synovitis of swollen joint(s) were not mentioned and could have made a difference between experts' opinions, even if this possibility seems low [18]. Indeed, one way to validate the proposition is to apply it to our current own patients in real life. This validation is ongoing with all of the panelists.

## Conclusions

According to the presented results, the STPR group recommends maintaining treatment without changing the molecule or the dose regimen when a patient with stable clinical status over the past 3 months has DAS28 ≤ 2.4. Above this threshold, the number of swollen joints should be considered - independently of or in addition to the DAS28 value - for the clinician to make a decision to maintain or to change therapy.

## Abbreviations

CI: confidence interval; DAS28: 28-joint disease activity score; DMARD: disease-modifying antirheumatic drug; ESR: erythrocyte sedimentation rate; OMERACT: Outcome Measures in Rheumatoid Arthritis Clinical Trials; PASS: patient acceptable symptom state; RA: rheumatoid arthritis; RAPID: routine assessment of patient index data; STPR: stratégie thérapeutique de la polyarthrite.

## Competing interests

The authors declare that they have no competing interests.

## Authors' contributions

All authors are members of the STPR group and thus participated in the project and answered interviews. MdB, BF and FG conceived of the project. FG performed the statistical analysis. MdB, BF XLL and FG organised the study. MdB and FG wrote the paper.

JFM, JMB, BC, R-MF, FL, OM, AS, and DW participated in data analysis, interpretation and assisted in manuscript preparation.

## Authors' information

STPR members who participated in the study are as follows: E Palazzo, G Streit, O Vittecoq, P Patoz, P Bennet, Y Maugars, Y Laborie, J Gillard, J Morel, MC Legouffre, H Cholvy, B Saint-Marcoux, S Lasbleiz, B De Bie, V Foltz, B Banneville, G Dubourg, P Philippe, F Pouyol, S Muller, H Korn, V Simon, C Collange, Ph Dieudé, M Ballard, Ch Best, S Jousse, J Allain, P Kervarec, B Augé, L Brault, Ch Piroth, F Pascaud, and N Gerard.
